# Adolescent idiopathic scoliosis: evaluating perioperative back pain through a simultaneous morphological and biomechanical approach

**DOI:** 10.1186/s12891-020-03462-4

**Published:** 2020-07-16

**Authors:** Maxime St-Georges, Alisson R. Teles, Oded Rabau, Neil Saran, Jean A. Ouellet, Catherine E. Ferland

**Affiliations:** 1 McGill Scoliosis and Spine Research Group, Montreal, Canada; 2 Shriners Hospitals for Children-Canada, 1003, boul. Décarie, Montreal, Quebec H4A 0A9 Canada; 3 Department of Experimental Surgery, McGill University, Montreal, Canada; 4 Integrated Program in Neurosciences, McGill University, Montreal, Canada; 5 Department of Surgery, Division of Orthopedics, McGill University, Montreal, Canada; 6 Department of Anesthesia, McGill University, Montreal, Canada

**Keywords:** Perioperative pain, Adolescent idiopathic scoliosis, 3D reconstruction, Spinal morphology, Postural balance

## Abstract

**Background:**

Adolescent idiopathic scoliosis (AIS) has been associated with diminished postural stability and a greater prevalence of back pain. Currently, the literature is lacking information on the effect of spinal fusion on both postural stability and its association with back pain. Our objectives were to evaluate the postsurgical effect of spinal morphological changes on static standing balance and assess the influence of these alterations on reported pain throughout the perioperative period.

**Methods:**

Twenty consecutive AIS patients schedule to undergo spinal fusion surgery were recruited and followed prospectively at the Shriners Hospitals for Children-Canada. Data was collected at the preoperative, 6 weeks and 6 months postoperative visits. Spinal morphology data was collected through 3D reconstructed simultaneous standing biplanar radiographs using the SterEOS software. Postural balance was assessed through Moticon© sensor insoles and analyzed through their software. The data was simultaneously collected as part of the Global Biomechanical and morphological Assessment. Pain was evaluated through self-reported questionnaires.

**Results:**

Morphological curve parameters were significantly reduced after surgery. Balance parameters did not change significantly throughout the perioperative period with the exception of the Center of Pressure of the left foot medial/lateral transient shift (*P* = 0.017) at 6 weeks. Of note, preoperative balance parameters were associated with the degree of right thoracic Cobb angles (*P* = 0.029 *R* = 0.528). Pain scores significantly improved 6 weeks and 6 months after the surgery. Pain intensity diminished in the thoracic and lumbar spine but worsen in the neck region at the 6 weeks and 6 months postoperative time points (*P* = 0.044). Greater residual Cobb angle difference between Mid thoracic and Thoracolumbar/Lumbar curves was associated with greater pain severity at 6 weeks postop (*P* < 0.005). In addition, greater residual thoracic deformity was associated with significant pain severity 6 months after surgery (*P* < 0.05).

**Conclusions:**

Improved spinal morphology of postsurgical AIS patients has no significant impact on their static standing balance. Suggesting that other factors apart from the spinal morphology may contribute to AIS patients’ balance during stance. Although balance did not influence pain severity, spinal morphology and its correction appear to have influenced the intensity and location of back pain.

## Background

The latest literature seems to agree that adolescents with idiopathic scoliosis have poorer balance control compared to adolescents without scoliosis [1–6]. Whether it is due to the spinal deformity itself, to a somatosensory alteration or a combination of both is yet to be confirmed [1, 2]. Studies exploring the association between the vestibular system and balance in patients with adolescent idiopathic scoliosis (AIS) found that when comparing them to a cohort of healthy controls, AIS patients depended significantly more on their vestibular and somatosensory systems to keep their balance [4, 7, 8]. It is hypothesized that neurophysiological compensations could result from the structural deformity of the spine [4], but also be the nature of the curve progression [1, 9]. It is therefore sensible to assume that spinal deformity correction would lead to changes in postural balance in AIS.

Corroborative evidence of this assumption has been previously reported. Valles et al. have shown significant improvement in objective parameters of postural control during quiet standing with both eyes opened and closed up to 1-year postoperatively in AIS patients [10]. However, contradictory findings have also been published. Schimmel et al. [11] have found that postural balance parameters 1 year after surgery did not differ from preoperative results. Similar results were obtained by other authors studying AIS [12, 13] and adults with spinal deformity [14]. However, since the reported literature is scarce and investigators have used different methodological approaches [10–15], it is difficult to draw a definitive conclusion on the subject.

It is also important to consider the consequences, such as back pain, of AIS caused spinal morphology and postural balance. In fact, back pain prevalence is higher in AIS patients compared to adolescents without scoliosis [16–20]. Despite recognizing that pain is a multifactorial disorder, most research focuses on its association between radiographic parameters [17, 19, 21, 22]. However, the literature lacks information on the influence of spinal morphology and postural balance on back pain in this population. Fortin et al. theorized that the biomechanical changes in the posture of AIS patients, creating a postural imbalance, could contribute to the onset of back pain [23]. In addition, despite most patients observing a reduction in baseline pain postoperatively [24], there is a subgroup of patients that either report an increased pain score or even develop chronic pain after surgery [25]. The association between postural balance according to spinal morphology and its effect on perioperative pain has not yet been investigated.

In order to evaluate the influence of morphology and postural balance on back pain, we designed a longitudinal study with AIS patients undergoing surgical treatment. Our objectives were to investigate the association between spinal deformity correction and postural balance changes and to evaluate its impact on postoperative back pain.

## Methods

### Population

Twenty (20) consecutive patients (15 females and 5 males) between the ages of 10 and 18 diagnosed with AIS and scheduled for a spinal fusion surgery were recruited at the Shriners Hospitals for Children - Canada. The mean age of the cohort was 14.9 ± 1.68 years, the average body mass index (BMI) was 20.66 ± 3.25 and the mean exposure time was 20.62 ± 2.93 s at the preoperative time point. Regarding to the curve type, there were (8) patients with Lenke 1, (5) Lenke 2, (2) Lenke 3, (1) Lenke 4, (2) Lenke 5, and (2) Lenke 6. Preoperative morphological results can be seen in Table 1. Patients were excluded of the study if their primary diagnosis was not AIS, could not take a standing radiograph, could not speak or understand French or English, had a cognitive disability preventing them from answering questionnaires, had previously undergone spinal or lower limb surgery or were diagnosed with neuromuscular impairment. Prior to study participation, written informed consent was obtained from all patients and if necessary, their parents. This project received ethical approval from McGill University (IRB# A08-M71-14B).

**Table 1:** Patients preoperative curve parameters

Patient	Lenke Type	TL/L Cobb angle(^**o**^)	MT Cobb angle(^**o**^)	PT Cobb angle(^**o**^)	Fusion Area
1	4	56.8	59.6		T2-L3
2	1	33.1	60.7	17.4	T3-L2
3	6	55.1	41.3		T3-L3
4	5	55.0	32.0	7.2	T5-L3
5	1	43.0	65.0		T2-L2
6	2	5.5	51.7	32.9	T4-T12
7	2	33.2	62.3	45.1	T4-L1
8	2	39.6	60.0	31.4	T3-L2
9	2	37.0	67.1	34.8	T3-T12
10	3	71.6	81.2	24.1	T4-L2
11	2	27.8	69.3	50.1	T4-L1
12	1	32.5	55.9	21.8	T4-L1
13	1	41.7	57.6	18.4	T3-L1
14	6	47.4	45.7	23.4	T4-L2
15	1	27.1	47.7	23.4	T3-L1
16	1	34.4	46.9	15.1	T4-T11
17	5	53.7	16.4	16.8	T11-L3
18	1	52.0	53.7	16.6	T4-L1
19	1	52.6	66.1		T4-L1
20	3	30.9	86.1	35	T2-L2

*TL/L* Thoracolumbar/lumbar, *MT* Main thoracic, *PT* Proximal thoracic, *T* Thoracic vertebra, and *L* Lumbar vertebra

### Global biomechanical and morphological assessment

Due to the lack of literature on simultaneous assessments of balance and spinal morphology an evaluation bias between the two can be observed. Thus, we created the Global Biomechanical and Morphological Assessment (GBMA). This simultaneous evaluation is comprised of a three-dimensional (3D) modelling assessment of the scoliotic spine and a balance assessment using wireless sensor insoles. These sensor insoles captured live data of the patient’s balance during their standing biplanar radiographs. A Cartesian plane was placed on the floor of the radiographic cabin as to associate the location and orientation of the feet to the spinal morphology. After the acquisition, the spinal morphology was reconstructed in 3D for better evaluation. The morphological data was then analyzed in parallel with the postural balance data to find associations. Since all variables are captured at the same time in the same position, this technique eliminates the bias of two separate evaluations of the balance and the spinal morphology while also providing a visual representation of their associations (as seen in the article by St-Georges et al. submitted to Scientific Reports). The GBMA was used at every time point during this study.

### Three-dimensional spinal structure assessment

Patients underwent simultaneous two-dimensional head-to-toe posterior-anterior (coronal) and lateral (sagittal) radiographs using the EOS-Imaging system (EOS-Imaging®, Paris, France) as part of the standard of care. This was done at every time point: preoperatively, 6 weeks and 6 months postsurgical. Patients were asked to place their hands on the wall in front of them to expose the spine without the obstruction of the humerus in the sagittal plane. This position also reduced the potential of a second exposure. Patients were asked to keep their eyes open and keep a normal breathing cycle not to disturb the image. Once the acquisition complete, all 60 spinal radiographs were reconstructed in three-dimension (3D) through the SterEOS software version 1.6.4.7977 (EOS-Imaging®, Paris, France) by trained professionals.

Radiological parameters extracted from the SterEOS software were: Cobb angles from the Proximal Thoracic (PT), Mid Thoracic (MT) and Thoracolumbar/Lumbar (TL/L) curves; the rotation in all three planes of these curves apical vertebrae (AVR); the intervertebral rotation in all planes of every vertebrae; the T1-T12 kyphosis; the L1-S1 lordosis; the pelvic incidence, axial rotation and tilt in the lateral and frontal planes; and the sacral slope. All of these variables were taken in the patient’s reference plane for time point comparison. The patient reference plane is a reconstruction based on all three-dimensional planes. It bases itself on the location of the acetabula inside the cabin to reconstruct the spine instead of the position of the patient, which changes from visit to visit [26]. This allows for better comparison through the perioperative period. Additional variables: torsion index of the main curve [27] and trunk shift were interpreted after the reconstruction. Visual description of the variables can be found in submitted work from St-Georges et al. 2019 to *Scientific Reports*.

### Balance assessment

The static standing balance assessment was conducted with Moticon© OpenGo sensor insoles (Moticon GmbH, Munchen, Germany) during the radiological capture. They were activated and then slipped under the patient’s feet in the cabin just prior to the radiographic exposure. The plantar recording was time matched with the radiograph acquisition to have a simultaneous picture of the effect of the abnormal spine on the biomechanics of the feet.

Every patient was sized for a pair of insoles. The patients were asked to stand comfortably with their feet pointing forward in the centre of the cabin. Once the patient entered the cabin and the insoles were slipped under their feet, their feet were marked upon the Cartesian plane. The patient’s feet were not moved nor reoriented to evaluate, as much as possible, their normal stance.

The insoles measured nine different balance parameters through 13 sensors in each insole. All parameters were based on the percentage of pressure or the centre of pressure (CoP) of each individual foot. These variables are as follows: the mean and standard deviation of the anteroposterior and mediolateral CoP location (CoP AP and CoP ML respectively), where a positive result is seen in the anterior and medial plane based on the zero located approximately at the metatarsal-phalangeal joints and in the midline of the foot; the CoP velocity, measured in mm/s; the CoP trace length, measured in meters; the CoP anteroposterior and mediolateral bounding box, where the greater the measurement the greater the CoP distance travelled in said plane; The CoP sway area, which is the product of both planes bounding box measurements; the mean foot pressure; and the pressure distribution in the front, mid and back of the foot. Moticon© OpenGo software version 01.11.00_11072-929d380 was used to analyze the balance variables at every time point. (Moticon GmbH, Munchen, Germany).

### Pain assessment

Self- reported questionnaires were given to the patient after their morphological and biomechanical assessment at each time point. The questionnaires given to the patients were: the Scoliosis Research Society Questionnaire-30 (SRS-30), and a modified version of the Adolescent Pediatric Pain Tool (APPT) containing a back diagram for pain location and intensity. The latter was evaluated with a 0–10 numeric rating scale where 0 is no pain and 10 is the worst pain imaginable. Pain severity groups were then created: mild (0–3), moderate (4–6) and severe (7–10). The questionnaires were asked to be done without the help of their parents to avoid bias. If for any reason the patient would have questions, the research assistant was at their disposal to answer them. All questionnaires were reviewed after completion and entered into a database.

### Statistical analysis

All statistical analyses were conducted using the software SPSS version 25 (IBM, Armonk, NY, USA). Descriptive and inferential statistics (mean and standard deviations) were used to display the patients’ demographic, morphological, balance and pain data. Paired student t-Tests were used to evaluate the differences between the preoperative and the postoperative time points for demographics, morphology, balance, and pain. Pearson correlations were done to estimate the association between 3D spinal structure and balance variables, and to assess the relationship between the morphological and pain-related parameters. A one-way ANOVA with Bonferroni Post-Hoc test was used to assess the significance between different pain severity groups. A Chi-square test was used to analyze the proportions of patients with pain throughout the perioperative period per painful back location. Significance for all evaluation was set at *P* < 0.005.

## Results

### Spinal morphological assessment

As seen in Table 2, all three (PT, MT, TL/L) Cobb angle means were significantly reduced whether 6 weeks or the 6 months after surgery compared to before (*P* < 0.001). The MT apical vertebra translation (*P* < 0.001), the TL/L apical vertebral rotation (6 weeks *P* = 0.029; 6 months *P* = 0.025), MT’s absolute torsion (6 weeks *P* < 0.001; 6 months *P* = 0.013) and the sacral slope (6 weeks *P* = 0.008; 6 months *P* = 0.002) also followed suit in their significant reduction at 6 weeks and 6 months postoperative. Others, MT apical vertebra rotation and L1-S1 lordosis were significantly reduced at 6 weeks but were no longer at 6 months postoperative. The T1-T12 kyphosis oppositely was significantly increased at 6 months but not at 6 weeks postoperative.

**Table 2:** Spinal morphology parameters – comparison between preoperative and postoperative timepoints

	Preoperative	6 weeks	*P*	6 months	*P*
TL/L Cobb angle (^o^)	41.50 ± 14.56	14.49 ± 7.331	< 0.001*	13.15 ± 8.27	< 0.001*
MT Cobb angle (^o^)	56.31 ± 15.83	18.95 ± 4.74	< 0.001*	18.13 ± 7.09	< 0.001*
PT Cobb angle (^o^)	25.85 ± 11.52	14.31 ± 7.76	< 0.001*	13.82 ± 8.92	< 0.001*
TL/L AVT (mm)	−11.1 ± 19.24	− 7.57 ± 11.58	0.366	− 5.15 ± 13.6	0.085
MT AVT (mm)	42.3 ± 18.76	−10.42 ± 10.66	< 0.001*	11.4 ± 13.38	< 0.001*
TL/L AVR (^o^)	7.35 ± 13.76	3.88 ± 8.27	0.029*	3.61 ± 9.94	0.025*
MT AVR (^o^)	−14.72 ± 8.96	− 9.06 ± 7.54	0.001*	−10.96 ± 9.43	0.060
PT AVR (^o^)	4.74 ± 7.46	0.61 ± 4.98	0.019*	3.99 ± 5.88	0.648
T1-T12 Kyphosis (^o^)	29.88 ± 13.95	32.49 ± 9.95	0.299	36.08 ± 10.02	0.023*
L1-S1 Lordosis (^o^)	57.02 ± 11.47	52.11 ± 9.64	0.005*	55.77 ± 10.43	0.534
MT Absolute Torsion (^o^)	33.46 ± 11.88	21.07 ± 9.03	< 0.001*	26.75 ± 11.94	0.013*
Lateral Pelvic Tilt (mm)	5.19 ± 3.52	5.74 ± 3.78	0.308	4.98 ± 4.06	0.664
Sagittal Pelvic Tilt (^o^)	6.21 ± 8.74	8.25 ± 8.95	0.079	6.76 ± 6.42	0.636
Pelvic Incidence (^o^)	51.07 ± 10.66	50.26 ± 9.65	0.118	48.45 ± 9.92	0.054
Sacral Slope (^o^)	44.85 ± 5.81	42.01 ± 4.93	0.008*	41.69 ± 6.07	0.002*

All results are shown as mean ± standard deviation. *TL/L* Thoracolumbar/lumbar, *MT* Main thoracic, *PT* Proximal thoracic, *AVT* Axial vertebral translation, *AVR* Apical vertebral rotation. A negative represents a translation towards the left for both apical translations, a left trunk shift and a right handed rotation when it comes to the apical vertebral axial rotation. *P* values are given in regards to the preoperative and their associated postoperative time point values. Paired Student’s t-test, * = significance was reached when *p* < 0.05

### Postural balance evaluation

All but one mean balance parameter did not differ significantly throughout the perioperative period (Table 3). The left foot medial/lateral location of the CoP was significantly different at 6 weeks after surgery (*P* = 0.017). Although when compared to the 6 months postoperative time point, no difference was observed with the preoperative results. Therefore, since no significant difference in postural balance was observed between the preoperative and the 6 months postoperative time points, no significant associations could be made with the changes in spinal morphology.

**Table 3:** Postural balance parameters – comparison between preoperative and postoperative time points

	Preoperative	6 weeks	*P*	6 months	*P*
Pressure left foot	46.50 ± 5.46	46.38 ± 6.58	0.938	44.98 ± 6.29	0.412
Pressure right foot	53.50 ± 5.46	53.62 ± 6.58	0.938	55.02 ± 6.29	0.412
Back feet pressure	60.08 ± 8.06	58.82 ± 12.82	0.584	62.45 ± 9.31	0.290
Mid-feet pressure	21.37 ± 5.31	22.05 ± 0.84	0.611	21.07 ± 7.76	0.854
Forefeet pressure	18.55 ± 4.81	19.13 ± 7.18	0.722	16.48 ± 6.40	0.202
Left anterior/posterior CoP	−46.80 ± 11.25	−42.06 ± 21.39	0.282	−52.45 ± 18.94	0.217
Left medial/lateral CoP	−2.51 ± 1..98	−1.33 ± 2.65	0.017*	−2.13 ± 3.43	0.547
Right anterior/posterior CoP	−47.55 ± 8.68	−45.21 ± 14.69	0.513	−48.89 ± 12.98	0.666
Right medial/lateral CoP	−2.53 ± 2.39	−3.38 ± 3.12	0.200	− 2.89 ± 2.92	0.732
Left foot CoP velocity	71.11 ± 29.61	71.91 ± 23.99	0.919	69.02 ± 22.46	0.723
Right foot CoP velocity	52.24 ± 13.57	54.69 ± 13.93	0.414	56.46 ± 12.44	0.230
Left foot CoP trace length	1.43 ± 0.51	1.44 ± 0.56	0.966	1.38 ± 0.53	0.739
Right foot CoP trace length	1.08 ± 0.36	1.09 ± 0.33	0.970	1.13 ± 0.32	0.706
Left foot sway area	23.99 ± 18.24	23.36 ± 13.59	0.873	24.95 ± 19.77	0.855
Right foot sway area	20.04 ± 24.71	14.87 ± 7.09	0.395	13.96 ± 5.33	0.295

All results are shown as mean ± standard deviation. Pressures are shown as proportions. Pressure units are in (N/cm2), Centre of Pressure (CoP) is measured in (mm), Velocity is measured in (mm/s), Trace Length in (m), and Sway Area in (mm2). *P* vales are given regards to the preoperative and their associated postoperative time point values. Paired Student’s t-test, * = significance was reached when *p* < 0.05

### Pain assessment

**Fig. 1:**
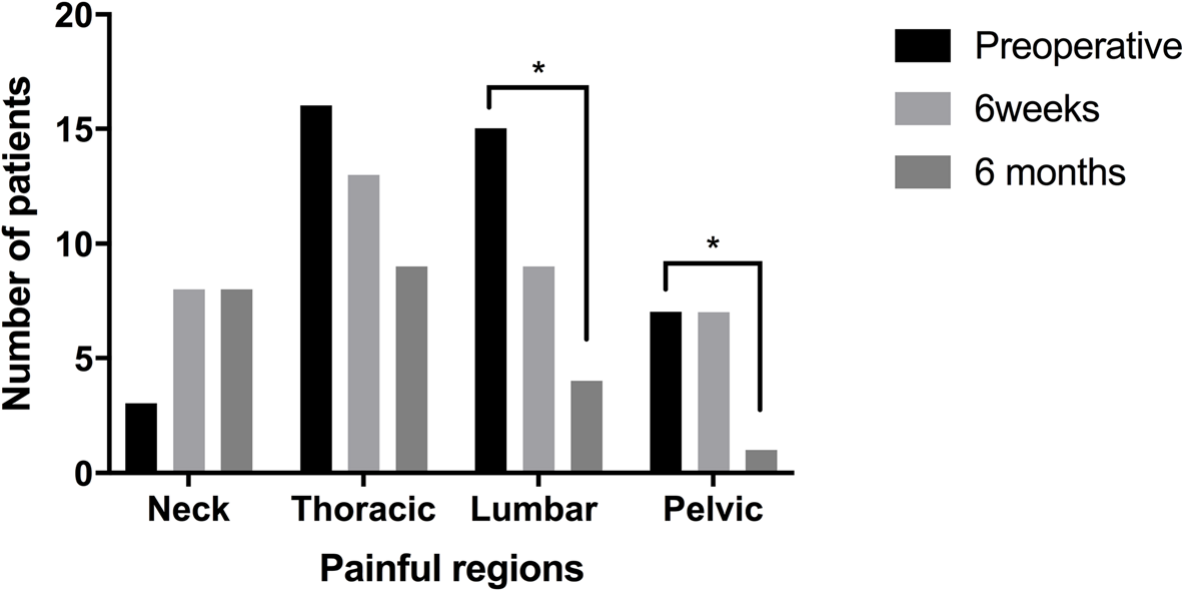
Percentage of patients with pain per location throughout the perioperative period. Note: Chi-square test, significant difference between proportion of patients that reported pain in the lumbar and pelvic area (*P* = 0.002 and *P* = 0.041 respectively) from preoperative baseline to 6 months postoperative

Perioperative pain data can be observed in Tables 4 and 5. When looking at the 6 weeks postoperative data only, pelvic pain (*P* = 0.265), and the number of painful regions (*P* = 0.625) were not significantly different. All APPT worst pain, thoracic and lumbar pain severity, and painful regions were reduced significantly after 6 months. These finding were also corroborated with the results of the SRS-30 Pain score as they improved significantly (*p* = 0.004) at 6 months posterior spinal fusion (PSF). In contrast, pain in the neck area was significantly worse (*P* = 0.044). Self-reported pain was significantly reduced in the lumbar and pelvic region (*P* = 0.002 and *P* = 0.041 respectively) after surgery (Fig. 1).

**Table 4:** Pain severity parameters – comparison between preoperative and postoperative time points

	Preoperative	6 weeks	*P*	6 months	*P*
APPT Worst Pain	4.85 ± 2.72	3.05 ± 2.33	0.027*	2.00 ± 2.00	< 0.001*
Neck Pain	0.30 ± 0.80	1.25 ± 1.92	0.043*	0.98 ± 1.53	0.044*
Thoracic Pain	3.50 ± 2.76	2.10 ± 2.32	0.042*	1.40 ± 1.96	< 0.001*
Lumbar Pain	3.30 ± 2.52	1.20 ± 1.61	0.005*	0.45 ± 0.95	< 0.001*
Pelvic Pain	1.63 ± 2.87	0.50 ± 1.00	0.265	0.33 ± 1.16	0.200
Painful Regions	1.90 ± 1.12	1.75 ± 1.07	0.625	1.10 ± 1.02	0.008*

All results are shown as mean ± standard deviation. All scores are based on a scale from 0 to 10, where 0 is no pain and 10 is the worst pain imaginable. Individual regional pain scores are based on the highest reported pain per area on a scale of 0–10. Painful regions are measured on a scale of 0 to 4, where 0 is no pain in any region and for every region where pain is felt there is the addition of 1 point. *P* values are given in regards to the preoperative and their associated postoperative time point values. Paired Student’s t-test, * = significance was reached when *p* < 0.05

**Table 5:** Proportions of patients with presence of pain in areas of the back

	Preoperative	6 weeks	6 months	*P*
Neck Pain	0.15	0.40	0.40	0.146
Thoracic Pain	0.80	0.65	0.45	0.070
Lumbar Pain	0.75	0.45	0.20	0.002*
Pelvic Pain	0.40	0.40	0.05	0.041*

All results are shown as proportions, *n* = 20. Pearson Chi-Square, *p* < 0.05, Paired Student’s t-test, * = significance was reached when *p* < 0.05

### Relationship between pain, spinal morphology and postural balance

#### Preoperative morphology and pain

**Fig. 2:**
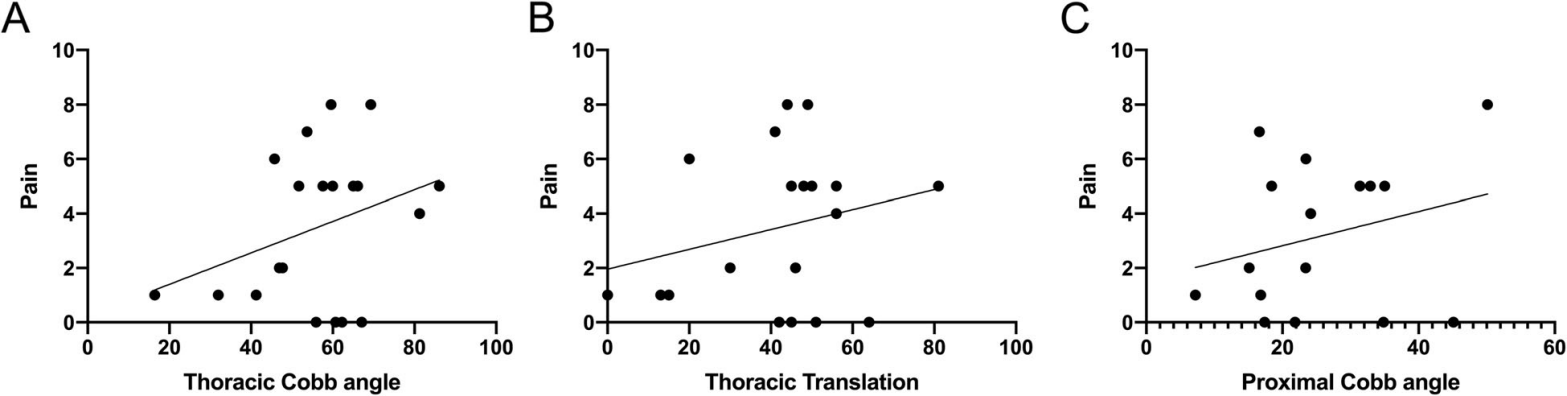
Preoperative thoracic pain severity and thoracic morphology. Note: Pearson Correlation, significant association between the severity of the pain in the thoracic area and the thoracic Cobb angle (*P* = 0.016; *R* = 0.593), thoracic apical vertebra translation (*P* = 0.030; *R* = 0.544) and the proximal thoracic Cobb angle (*P* = 0.018; *R* = 0.665)

As seen in Fig. 2 at the preoperative time point several associations were found when looking at the location of the pain and thoracic parameters. A moderate correlation was found between the severity of the thoracic Cobb angle and the severity of the pain (Fig. 2a) (*P* = 0.016; *R* = 0.593) and the severity of the right thoracic translation (Fig. 2b) (*P* = 0.030; *R* = 0.544). A strong correlation (*P* = 0.018; *R* = 0.665) was seen between the severity of the proximal thoracic Cobb angle and the severity of the pain (Fig. 2c).

#### Six weeks postoperative morphology and pain

**Fig. 3:**
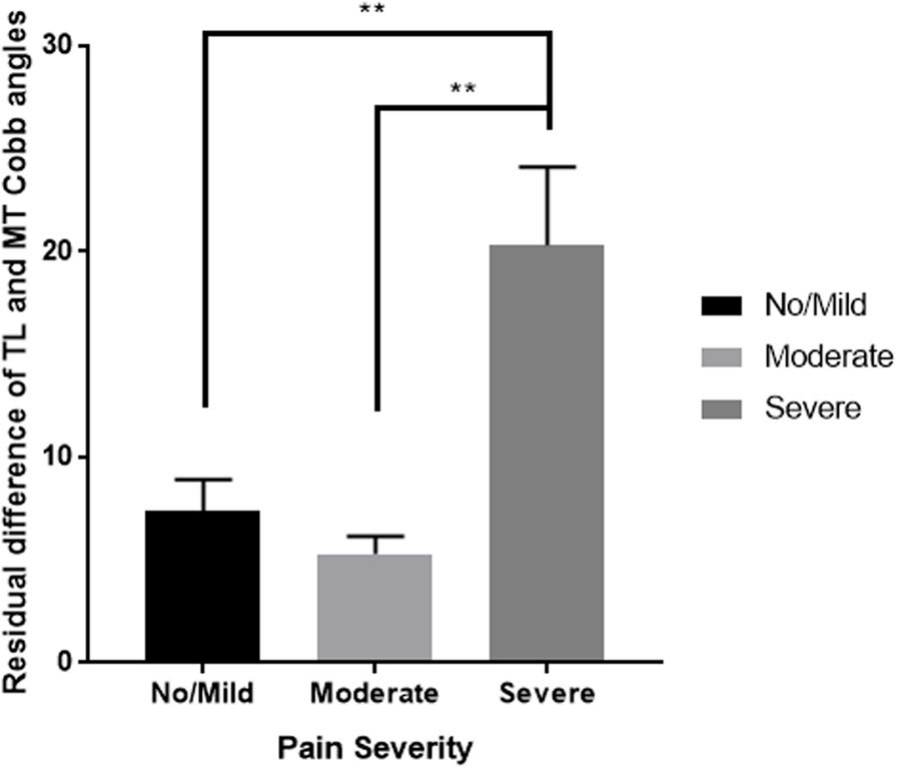
Six weeks postoperative pain and residual deformity. Note: One-way ANOVA, **P < 0.005, significant difference between the severity of the pain felt and the residual difference between the thoracolumbar/lumbar (TL) and the main thoracic (MT) Cobb angles. *P* = 0.004 between No/Mild pain and Severe pain. *P* = 0.001 between Moderate and Severe pain

Six weeks after the surgical intervention, the difference between the lumbar and thoracic residual Cobb angles was associated with the pain severity reported (Fig. 3). It suggests that the greater the difference between the two residual curves the more likely the patient is to have severe pain. An association was also found between the SRS-30 Pain score and the thoracic translation (*P* = 0.040; *R* = 0.463).

#### Six months postoperative morphology and pain

**Fig. 4:**
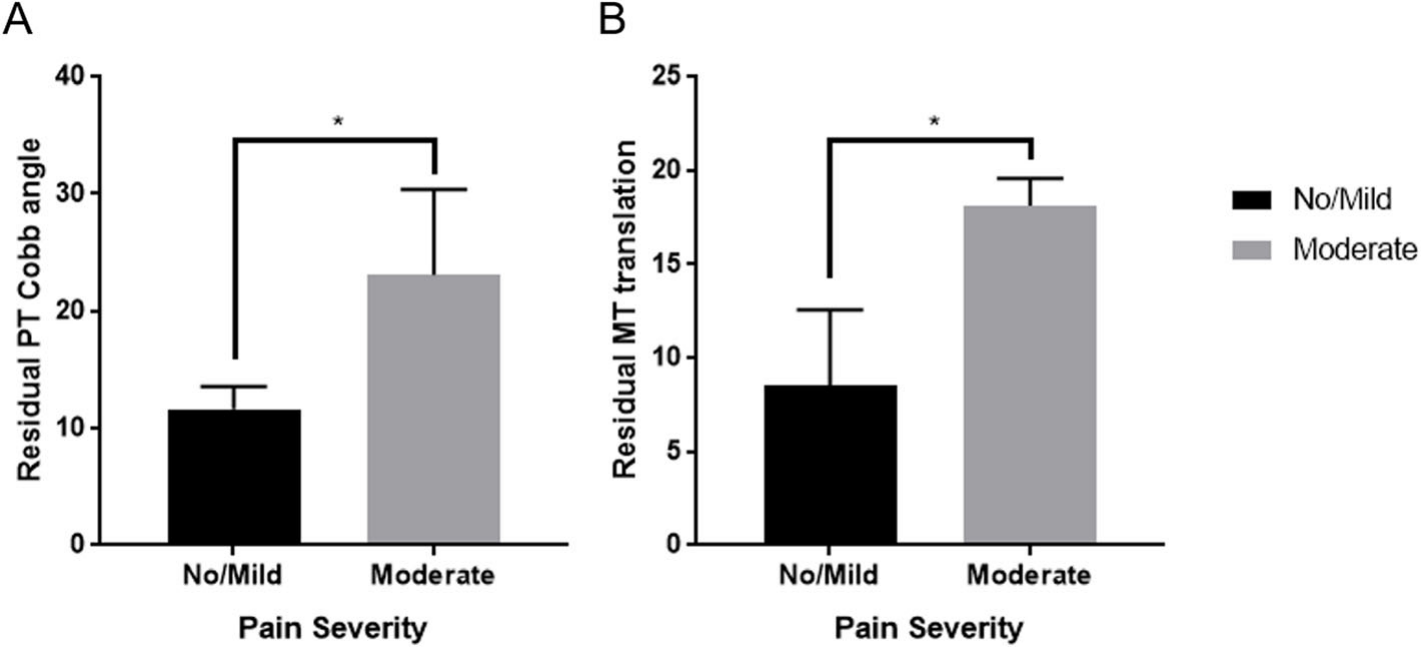
Six months postoperative pain and thoracic residual deformity. Note: Independent Student’s *t*-Test, *P < 0.050, significant difference between the pain severity and the residual proximal thoracic Cobb angle (*P* = 0.032) and the residual thoracic translation (*P* = 0.041)

At 6 months post PSF, an association between the residual thoracic area deformity and the pain severity reported was identified (Fig. 4). It shows that patients who tend to have a greater residual PT curve and/or thoracic apical vertebra translation tend to report greater severity of pain (*P* = 0.032 and *P* = 0.041 respectively). Another significant association emerged when looking at the reported severity of the pain and lumbar translation: the apical translation of the lumbar spine was also associated with a greater pain severity (*P* = 0.023).

#### Perioperative balance and pain

**Fig. 5:**
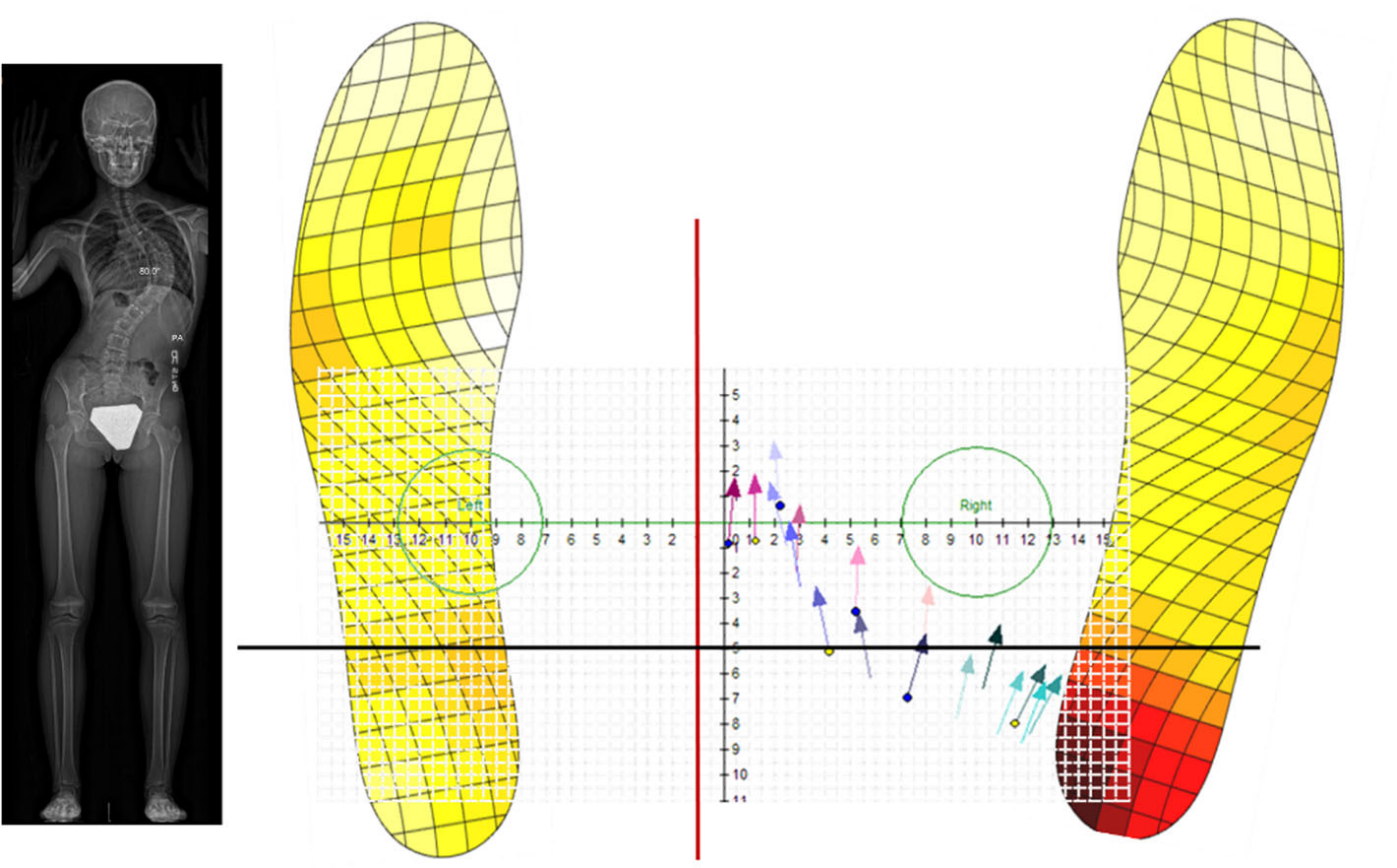
Preoperative right trunk shift and balance. Note: Pearson Correlation, significant association between degree of right main curve Cobb angle and amount of pressure felt in the right foot preoperatively. *P* = 0.029 *R* = 0.528

Regarding postural balance and pain, an association between the trace length of the CoP of the right foot and the SRS-30 pain score (*P* = 0.009; *R* = 0.569) was observed at 6 months. Also, while comparing patients that reported no/mild and moderate pain, patients that fell in the latter applied more pressure on the back of their feet (*P* = 0.050). Balance parameters were also associated with the degree of right thoracic Cobb angles at the preoperative time point (*P* = 0.029 *R* = 0.528) (Fig. 5). No other association with the patients’ mean balance parameters, whether morphological or painful, was found perioperatively.

## Discussion

This study presents a novel comprehensive evaluation, the GBMA, simultaneously assessing the spinal morphology and postural balance of AIS patients. It uses state-of-the-art 3D reconstruction of biplanar radiographs to examine the spinal morphology variables in all three planes while also using the wireless capacity of sensor insoles to concurrently evaluate postural balance. The results presented in this study concerning our first research question indicate that objective balance parameters have not changed post-operatively despite the realignment of the spine. Thus, suggesting no association between the change in morphology and the postural balance. These results, contradict our hypothesis but are supported by some of the literature on the subject [11–14, 28]. However, our second hypothesis was confirmed. Overall postoperative pain severity, pain in the thoracic and lumbar area, and the number of painful regions diminished significantly 6 months after surgery. In addition, proportional pain intensity has diminished significantly in the lumbar and pelvic area as well as a trend towards significance in the thoracic region 6 months after surgery. These results support the fact that surgery does reduce the pain felt by patients [24]. One could infer that spinal fusion reduces the spinal morphological malalignment, which could be responsible for the preoperative spinal pain, and that if not corrected patients are at higher risk of having ongoing back pain. Our data also identified several biomechanical and morphological variables associated with perioperative pain confirming our hypothesis stating that the PSF spinal modifications would produce a reduction in reported pain.

### Perioperative changes in morphology and their association to balance

Our study reports no significant difference in balance in the perioperative period in patients with AIS. This would suggest that the change of the spinal morphology would not affect the balance in this short postoperative period of time. This might be due to the lack of time for the body to fully adapt to the new posture. Patients were used to the way they stood before surgery and it possibly takes a longer period to adapt its balance to the new spinal morphology. Schimmel et al. [11], de Abreu et al. [28], and O’Beirne et al. [13] have also demonstrated that balance in AIS patients does not change with the modification of the spinal alignment up to a year after surgery. However, Valles et al. [10] have demonstrated that patients with AIS had a significantly better postural balance with their eyes closed, and significantly worst postural balance with their eyes opened. They also could significantly better regulate their balance with constant feedback during quiet standing at 1 year postoperatively [10]. In spite of the above results, we have found an association between the amount of pressure exerted on the right foot and right thoracic deformity preoperatively, but not postoperatively. Suggesting a relationship between the morphology and the balance before the surgical intervention that plays a lesser role postoperatively. Subsequent studies should consider including a longer follow-up period to confirm these results. The lack of significant associations could be explained by one of the leading theories regarding the disparity in balance of AIS patients. It theorizes that AIS patients’ decreased balance could be associated with an intrinsic impaired dynamic regulation of sensory-motor integration effecting the patient’s balance [1, 8, 29, 30]. Thus, suggesting that the morphology might not be the main root of the balance issues in these patients and why we were not able to see a change throughout the perioperative period.

These reported results, for the exception of an association between the severity of the pain and pressure in the back of the feet at 6 months post-surgery, also suggest that since the mean postural balance does not change throughout the perioperative period it has little effect on the pain felt by the patient. de Abreu et al. [28] evaluated pain in these patients using the SRS questionnaire and although they infer that the slight decrease in oscillation could be a reason for a decrease in pain they lacked to show any significant associations, thus aligning with our conclusions.

### Perioperative report of pain and its associations

In accordance with previous literature, our results highlight a decrease in pain severity after PSF and they also identify spinal morphological variables associated with the pain felt throughout the perioperative period. This, in part, confirms our second hypothesis, for which the morphology does impact the pain felt. The decrease in the severity of pain compliments the works of authors such as Sieberg et al. [24], Merola et al. [30], Connelly et al. [31] and Mimura et al. [32] that identified a significant decrease in pain reported after PSF in AIS patients as early as 6 months and as late as 5 years.

Interestingly, we identified that self-reported pain prevalence (Table 5 and Fig. 1) and pain intensity (Table 4) in the neck area increased from the baseline to the post-operative assessments. One possible explanation to increased pain in the neck area is the surgical trauma to the upper thoracic spine and its musculature in patients in which fusion was extended to that area. Another hypothesis is a possible influence of reciprocal changes in cervical spinal alignment after surgical correction. Further larger studies should explore pain referred to the neck area after surgical correction in AIS.

When considering our independent time point results, they indicate moderate to strong correlations between the thoracic deformity and the pain felt in the thoracic area before surgery. Recent literature [19, 33], found similar results, associating severity and the location of the curve in the thoracic area to the pain reported in said area. In all cases, the association was made at the preoperative time point but does not seem to persist throughout the postoperative period. Future studies should explore this concept and validate these results with a greater cohort permitting them to subgroup more accurately.

At 6 weeks and 6 months post PSF, our results show an association between the residual curves of the spine and the pain felt. To our knowledge, this is the first time this has been reported. Although the fusion of the lumbar vertebrae is usually avoided unless necessary to permit the flexibility of the spine, results presented in this study suggest that not ensuring stability in the lumbar region by diminishing the lumbar translation and reducing the lumbar AVR, creating a greater difference between the severity of the residual lumbar and thoracic curves, could be detrimental to the pain felt in the early postsurgical period. This association is not observed at 6 months, thus suggesting that it might only be a result of acute postsurgical pain. This might explain why authors such as Mimura et al. have not observed these effects later on in the postoperative period. We hypothesize that this might be due to the sudden uneven adaptation of muscle activation in the lower back for support creating a strenuous pain. Future research should explore the difference in the early postsurgical report of pain and last vertebrae fused. The 6 months postoperative association indicates a relationship between residual thoracic deformity and pain severity. Suggesting, that if the initial deformity is not corrected properly, it could result in greater severity of pain and possibly in its chronicity. Future studies should validate these results with greater sized cohorts. Although, pain is multifactorial [34] and biomechanical and morphological factors account for only a portion of the explanation of pain reported [27], these results, when and if validated, could help improve the pain management of patients through the perioperative period. This could be done by rethinking the surgical planning, potentially making improvements to the lumbar or the thoracic correction but also by identifying patients at risk of more severe pain and adapting their management accordingly.

### Limitations and strengths

This study has limitations that should be taken in consideration. The small sample size restricts the power of the analysis and diminishes the possibility of subgrouping by type of curves. Therefore, the evaluation of the effect of different curvatures on spinal balance was restrained. Evaluating patients with a right thoracic curve and patients with both right thoracic and left lumbar curves as part of the same cohort with possibly different balance [35], could have affected the mean variables of balance measured. Future studies should research the effect of independent subgrouping of curves and their effect on balance throughout the perioperative period thus eliminating the aforementioned possible bias. As per hospital protocol, during the radiological acquisition, to not obstruct the image of the sagittal spine, patients were asked to put their hands on the wall, without standardizing their hand pressure. This could have affected the postural balance parameters of the patients [36], especially the CoP variables in the anteroposterior plane. Future studies should try to evaluate patients with an in-cabin posture that does not require the support of the hands [36]. Although the insoles used in this simultaneous evaluation have been showed to identify overall comparable balance data to a force plate in the article under review by *Spine* from St-Georges et al. 2020, they do lack specificity in CoP displacement in static standing evaluation [37–39]. Therefore, the insoles might not have captured subtle differences in balance, possibly causing the lack of balance difference observed in the perioperative period. That said, these were able to be used simultaneously during radiological acquisition, future studies should consider their involvement in overall clinical analysis. These patients were followed up to 6 months post-surgically, it might not have been enough time to examine a change in postural balance. Future studies should gather data on a longer period of time to examine balance change through time due to the new spinal alignment. Future work should continue utilizing GBMA for its strength in assessing the morphology and plantar biomechanics simultaneously as to better understand their relationship as well as their possible effect on pain.

To our knowledge, this was the first study of its kind to evaluate simultaneous 3D standing biplanar radiographs at the same time as postural balance to understand pain. This methodological strength permits the assessment of the effect of the 3D morphology of the spine on balance without reducing the direct correspondence that comes with different evaluations. Which also present a stronger morphological and biomechanical profile to associate to the perioperative pain report of the patient.

## Conclusion

Spinal morphology in AIS influences pressure distribution across patients’ feet preoperatively. However, balance parameters do not change throughout the perioperative period, suggesting that other factors beyond the spinal morphology could contribute to AIS patients’ balance during stance. Although balance shows no association to pain, spinal morphology and its correction appears to influence the severity and location of back pain.

## Data Availability

The datasets used/analysed during the current study are available from the corresponding author upon reasonable request.

## Abbreviations

3D: Three-dimensional
AIS: Adolescent idiopathic scoliosis
APPT: Adolescent Pediatric Pain Tool
AVR: Apical Vertebrae Rotation
BMI: Body mass index
CoP: Centre of Pressure
CoP AP: Centre of Pressure Anteroposterior
CoP ML: Centre of Pressure Mediolateral
GBMA: Global Biomechanical and Morphological Assessment
MT: Mid Thoracic
PSF: Posterior spinal fusion
PT: Proximal Thoracic
SRS-30: Scoliosis Research Society Questionnaire-30
TL/L: Thoracolumbar/Lumbar
